# Neuropeptide Y prolongs non-social memory and differentially affects acquisition, consolidation, and retrieval of non-social and social memory in male mice

**DOI:** 10.1038/s41598-017-07273-x

**Published:** 2017-07-28

**Authors:** Johannes Kornhuber, Iulia Zoicas

**Affiliations:** Department of Psychiatry and Psychotherapy, University Hospital, Friedrich-Alexander-University Erlangen-Nuremberg, 91054 Erlangen, Germany

## Abstract

Neuropeptide Y (NPY) and its receptors (especially Y1, Y2, and Y5) are highly expressed in brain regions involved in learning and memory processes. Accordingly, NPY was shown to modulate cognitive functions in rodents. Here, we investigated possible memory-enhancing effects of NPY and determined the role of the NPY system in the acquisition, consolidation, and retrieval of non-social and social memory in mice, using the object and social discrimination tests, respectively. Intracerebroventricular (icv) infusion of NPY (1 nmol/2 µl) prolonged retention of non-social (object) memory, but not of social memory. This effect was blocked by the Y1 receptor antagonist BIBO3304 trifluoroacetate (2 nmol/2 µl), but not by the Y2 receptor antagonist BIIE0246 (2 nmol/2 µl). While icv infusion of NPY did not affect the acquisition, consolidation, and retrieval of non-social and social memory, icv infusion of BIBO3304 trifluoroacetate and BIIE0246 blocked the consolidation of non-social memory and the retrieval of both non-social and social memory. This study suggests that NPY has memory-enhancing effects in a non-social context by specifically acting on Y1 receptors. It further suggests that the central NPY system exerts differential effects on the sequential phases of non-social and social memory.

## Introduction

Memory is defined as the ability of the brain to encode, store, retain, and subsequently recall information and past experiences and can be classified according to function (working or reference memory), content (implicit or explicit memory), duration (short- or long-term memory), and nature (associative or non-associative memory)^1, 2^. Memory can also be classified according to the sequential phases involved, including acquisition, consolidation, retention, and retrieval of information. In simplistic terms acquisition can be defined as the ability to learn from new experiences, consolidation is solidification of the new information for storage, retention is the preservation of the information, and retrieval is the later recall of the information^2^. Translating the behavioral observations after pharmacological interventions into single memory phases is not always straightforward. Therefore, when substances are applied before training they mainly affect acquisition, after training they mainly affect consolidation and retention, and before testing of previously acquired memory they mainly affect retrieval. However, some overlapping situations can occur. For example, substances applied before training might exert effects lasting long enough to affect subsequent processes like consolidation^2^.

The electro-chemical signaling which occurs within neuronal networks and is undertaken by neurotransmitters, including neuropeptides, is necessary for learning and memory processes. One of the most abundantly expressed neuropeptides in the mammalian brain is neuropeptide Y (NPY), a 36-amino-acid peptide which exerts its biological effects through five subtypes of G_*i*_-protein-coupled receptors termed Y1, Y2, Y4, Y5, and y6^3^. Y1, Y2, and Y5 receptors are the most prominent in the brain and are expressed in regions involved in learning and memory processes, such as the hippocampus, amygdala, cingulate cortex, thalamus, hypothalamus, and cerebral cortex^4^. NPY and its receptors regulate important biological and pathophysiological functions, such as blood pressure, neuroendocrine secretions, seizures, neuronal excitability, and neuroplasticity^5–10^. NPY also causes a variety of behavioral effects, i.e. stimulates food intake^5^, promotes social interaction by acting on Y1 and possibly on Y2 receptors^11, 12^, and has anxiolytic and antidepressant-like effects by acting mainly on Y1 and Y2 receptors, respectively^13, 14^. An increasing number of studies indicate a role of NPY in learning and memory and have shown that NPY exerts both inhibitory and stimulatory effects depending on the type of memory, sequential phase, dose applied, receptor subtype, and brain region. As such, NPY was shown to impair acquisition in cued and contextual fear conditioning by acting on Y1 receptors^15–17^, and to impair retention and retrieval of fear memories presumably by synergistically acting on Y1 and Y2 receptors^18–20^. NPY is thereby acting as a resilience factor against exaggerated fear responses after stress and adverse events. In operant conditioning tasks, such as passive and active avoidance tests, however, NPY was shown to enhance memory consolidation, retention, and retrieval^21, 22^, but did not affect acquisition^23^. The effects of NPY on memory retention appear to be region-dependent since NPY enhanced retention when infused into the rostral hippocampus and septum, impaired retention when infused into the amygdala and caudal hippocampus, and was ineffective when infused into the thalamus, caudate, or cortical regions above the rostral hippocampus and septum^24^. NPY was also shown to counteract the amnesic effects of the protein synthesis inhibitor anisomycin and the muscarinic receptor antagonist scopolamine^21^, and to attenuate learning impairments induced by the non-competitive NMDA receptor antagonist dizocilpine (MK-801)^25^.

Although the involvement of NPY in learning and memory has been repeatedly demonstrated, it is unclear whether NPY can prolong the retention of memories. Furthermore, no studies to date investigated the involvement of the NPY system in social memory. Therefore, we first investigated for how long are non-social and social memory retained in our experimental protocols, and assessed whether NPY can prolong the retention of memory in the object and social discrimination tests^26, 27^. Next, we investigated whether NPY and its Y1 and Y2 receptors (given their role in social behavior, learning, and memory) are involved in the acquisition, consolidation, and retrieval of non-social and social memory.

## Materials and Methods

### Animals

Male CD1 mice (Charles River, Sulzfeld, Germany, 10 weeks of age) were individually housed for 1 week before experiments started and remained single-housed throughout the experiments. Sex-matched 3-week-old juvenile CD1 mice were used as social stimuli for the social discrimination test. Mice were kept under standard laboratory conditions [12:12 light/dark cycle, lights on at 06:00 h, 22 °C, 60% humidity, maintenance food for rat/mouse (ssniff Spezialdiäten GmbH, Soest, Germany) and water *ad libitum*]. Experiments were performed during the light phase, between 09:00 and 16:00, in accordance with relevant guidelines and regulations, and approval from the Committee on the Ethics of Animal Experiments of the Government of Mittelfranken. All efforts were made to minimize animal suffering and to reduce the number of animals used.

### Stereotaxic cannula implantation

Implantation of the guide cannula (21 G, 8 mm length; Injecta GmbH, Germany) for intracerebroventricular (icv) infusions was performed under ketamine-xylazine anesthesia (intraperitoneal injection of 120 mg/kg Ketavet and 16 mg/kg Rompun, respectively) as previously described^28–30^, 2 mm above the right lateral ventricle (from Bregma: +0.2 mm, lateral: +1.0 mm, depth: +1.4 mm). After surgery, mice were handled for 5 days before experiments started.

### Intracerebral infusions

Mice received manual icv infusions of either vehicle (Veh; distilled H_2_O; 2 µl), porcine NPY (1 nmol/2 µl; PeptaNova, Sandhausen, Germany), selective Y1 receptor antagonist BIBO3304 trifluoroacetate (BIBO; 2 nmol/2 µl; Tocris Bioscience, Bristol, UK; displays >2600-fold selectivity over Y2, Y4, and Y5 receptors), or Y2 receptor antagonist BIIE0246 (BIIE; 2 nmol/2 µl; Tocris Bioscience, Bristol, UK; displays >650-fold selectivity over Y1, Y4, and Y5 receptors) via an infusion cannula (23 G, 10 mm length) inserted into the guide cannula and connected via polyethylene tubing to a Hamilton syringe. The infusion system was left in place for 30 s following the infusion to allow diffusion of the solution.

The correct infusion site was histologically verified; accordingly, all guide cannulas were implanted correctly and no mice were excluded from the study. NPY, BIBO, and BIIE doses and timing of administration were selected based on previous studies^13, 16, 28, 29^.

### Behavioral paradigms

Mice were tested in the object discrimination test one week after surgery and in the social discrimination test one week later^26, 27, 31, 32^. Treatment was counterbalanced between the tests to minimize possible confounding effects^31, 32^.

### Object discrimination test

The ability of mice to discriminate between a previously encountered (*same*) and a *novel* object was tested in the object discrimination test^26, 27^, which was adapted to be comparable to the social discrimination test described below. An unknown object (*same*) was placed in one corner of the home cage of the experimental mouse for 4 min (acquisition period). After a defined inter-exposure interval (IEI), i.e. 1 h, 4 h, or 6 h, the *same* object was reintroduced along with a *novel* object for additional 4 min (discrimination period). The *same* object was placed at the same location as during the acquisition period. Several plastic objects that differed in color (pink, green, orange), shape (flower, diamond, square), and size (3–4.5 cm × 3 cm × 1 cm) were used. Objects were balanced between groups to prevent for possible object preference. Objects were cleaned between trials with water containing a small amount of detergent (Manisoft; Ecolab Deutschland GmbH). Experiments were recorded and the time spent investigating the objects (sniffing/touching) was analyzed by an observer blind to the treatment condition using JWatcher (Version 1.0, Macquarie University and UCLA). To verify the reliability of the blind observer, 10% of videos (chosen randomly) were analyzed twice; this method revealed less than 3% inter-analysis variability. The percentage of time investigating the *same* and the *novel* object (time investigating *same* or *novel* object/time investigating *same* + *novel* object × 100%) during the discrimination period was calculated. A higher investigation time directed toward the *novel* versus the *same* object indicated object discrimination and intact object memory.

### Social discrimination test

The ability of mice to discriminate between a previously encountered (*same*) and a *novel* conspecific was tested in the social discrimination test as previously described^26, 27^. A sex-matched juvenile mouse was introduced in the home cage of the experimental mouse for 4 min (acquisition period). After a defined IEI, i.e. 1 h, 2 h, or 4 h, the *same* juvenile was reintroduced along with a *novel* juvenile for additional 4 min (discrimination period). Shorter IEI were used for the social discrimination test, as it was previously shown that object memory is retained longer than social memory in rats^31^. Experiments were recorded and the time spent investigating the juvenile mice (sniffing the anogenital and head/neck regions) was analyzed by an observer blind to the treatment condition using JWatcher. As for the object discrimination test, 10% of videos (chosen randomly) were analyzed twice and revealed less than 3% inter-analysis variability. The percentage of time investigating the *same* and the *novel* juvenile mouse (time investigating *same* or *novel* mouse/time investigating *same* + *novel* mouse × 100%) during the discrimination period was calculated. A higher investigation time directed toward the *novel* versus the *same* juvenile indicated social discrimination and intact social memory. The 3-week-old juvenile mice did not elicit play or aggressive behaviors.

### Statistical analysis

For statistical analysis, PASW/SPSS (Version 21) was used. Object and social discrimination behavior (expressed as percentage) were analyzed by two-way ANOVA for repeated measures (factors stimulus × IEI or factors stimulus × treatment), followed by a Bonferroni’s post-hoc analysis whenever appropriate. Discrimination behavior expressed as duration in sec was also analyzed and revealed similar results as when expressed as percentage. Total investigation times between groups during the acquisition period were compared using one-way ANOVA (factor group or factor treatment) and revealed no significant differences. Statistical significance was set at p < 0.05. Overall statistics are shown in Table 1.

**Table 1:** Overall statistics for non-social and social memory as presented in Figs 1–6.

	Stimulus effect	Stimulus × IEI effect
Fig. 1A	F(1,42) = 111.5; p < 0.001*	F(2,42) = 44.59; p < 001*
Fig. 1B	F(1,42) = 31.40; p < 0.001*	F(2,42) = 5.89; p = 0.006*
	**Stimulus effect**	**Stimulus × treatment effect**
Fig. 2A	F(1,30) = 8.57; p = 0.006*	F(1,30) = 16.75; p < 0.001*
Fig. 2B	F(1,20) = 0.06; p = 0.81	F(1,20) = 1.79; p = 0.20
Fig. 3	F(1,68) = 19.23; p < 0.001*	F(5,68) = 3.97; p = 0.003*
Fig. 4A	F(1,64) = 109.0; p < 0.001*	F(3,64) = 0.03; p = 0.99
Fig. 4B	F(1,62) = 141.3; p < 0.001*	F(3,62) = 0.12; p = 0.95
Fig. 5A	F(1,56) = 25.97; p < 0.001*	F(3,56) = 7.26; p < 001*
Fig. 5B	F(1,88) = 121.6; p < 0.001*	F(3,88) = 0.72; p = 0.55
Fig. 6A	F(1,64) = 34.52; p < 001*	F(3,64) = 7.72; p < 0.001*
Fig. 6B	F(1,60) = 87.53; p < 0.001*	F(3,60) = 20.56; p < 0.001*

IEI, inter-exposure interval. Stimulus effect refers to the *novel* and the *same* object and to the *novel* and the *same* juvenile. Two-way ANOVA for repeated measures followed by Bonferroni post-hoc test; *p < 0.05.

## Results

### Non-social memory is retained for at least 4 h, whereas social memory for at least 2 h

**Figure 1:**
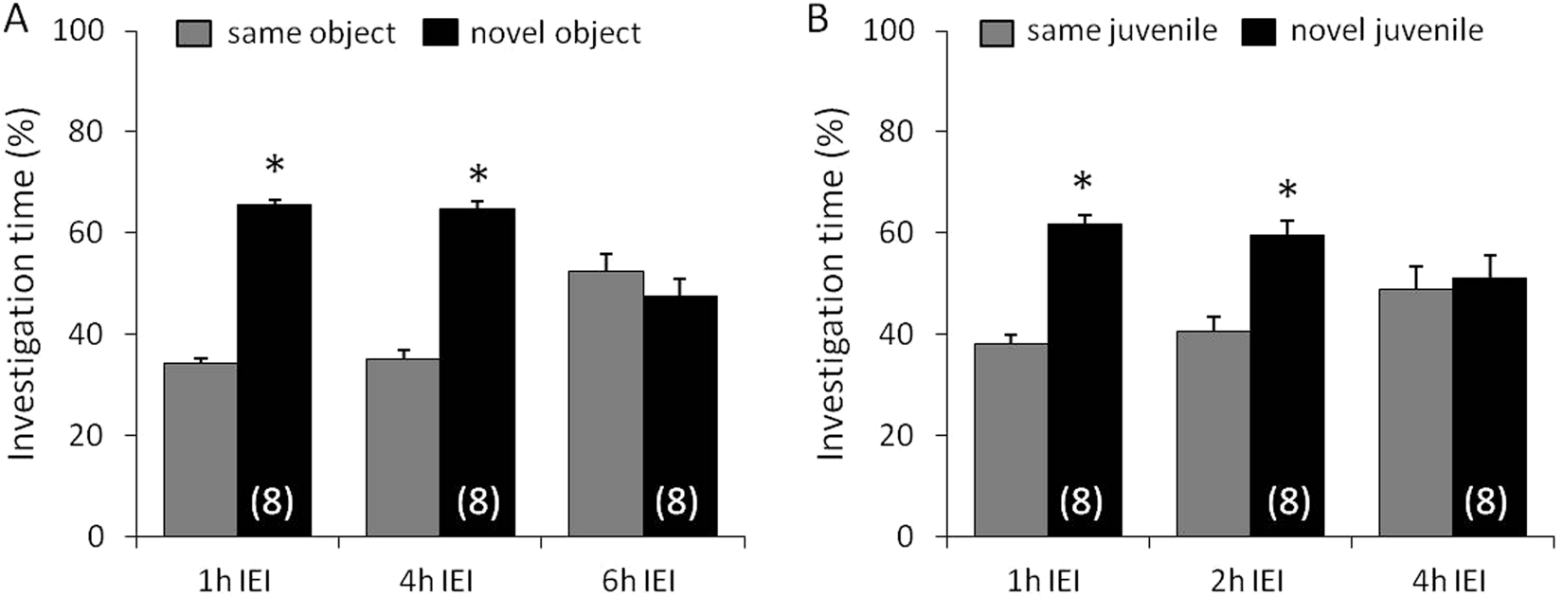
Non-social memory is retained for at least 4 h, whereas social memory for at least 2 h. Percentage investigation of the *same* and the *novel* object (**A**) or juvenile (**B**) during the discrimination period of the object and social discrimination test, respectively. Intact memory and discrimination abilities are reflected by an increased investigation of the *novel* versus the *same* stimulus after a defined inter-exposure interval (IEI). Data represent means + SEM, and numbers in parentheses indicate group sizes. *p < 0.05 versus *same* stimulus.

To determine for how long mice are able to remember a non-social and a social stimulus, mice were tested in the object and social discrimination test, respectively, with variable IEI between the acquisition and discrimination period of the tests. Untreated mice showed a higher investigation of the *novel* versus the *same* object after an IEI of 1 h and 4 h, reflecting object discrimination and intact non-social memory (p < 0.05, Fig. 1A). After an IEI of 6 h, however, mice showed similar investigation of the *same* and the *novel* object, reflecting impaired non-social memory (Fig. 1A). After an IEI of 1 h and 2 h, mice showed intact social memory (p < 0.05, Fig. 1B), whereas after an IEI of 4 h social memory was impaired.

### NPY prolongs retention of non-social, but not of social, memory

**Figure 2:**
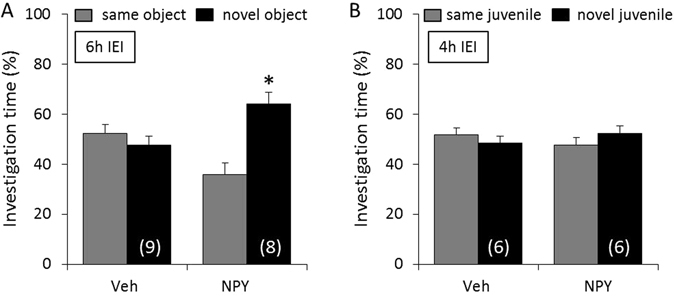
Neuropeptide Y (NPY) prolongs retention of non-social, but not of social, memory. Mice were infused icv with either vehicle (Veh; 2 µl) or NPY (1 nmol/2 µl) immediately after the acquisition period of the object (**A**) and social (**B**) discrimination test, respectively. Intact memory and discrimination abilities are reflected by an increased investigation of the *novel* versus the *same* stimulus after an inter-exposure interval (IEI) of 6 h (**A**) or 4 h (**B**). Data represent means + SEM, and numbers in parentheses indicate group sizes. *p < 0.05 versus *same* stimulus.

To determine whether NPY can prolong the retention of non-social and social memory, mice were infused icv with either Veh or NPY immediately after the acquisition period of the object and social discrimination test, respectively. To test for possible memory-enhancing effects of NPY, IEI of 6 h and 4 h were selected for the object and social discrimination test, respectively. Whereas Veh-treated mice showed impaired non-social memory after an IEI of 6 h, NPY-treated mice showed a higher investigation of the *novel* versus the *same* object, reflecting object discrimination and intact non-social memory (p < 0.05, Fig. 2A). In contrast, both Veh- and NPY-treated mice showed impaired social memory after an IEI of 4 h, reflected by similar investigation of the *same* and the *novel* juvenile (Fig. 2B).

### NPY prolongs retention of non-social memory by acting on Y1, but not on Y2, receptors

**Figure 3:**
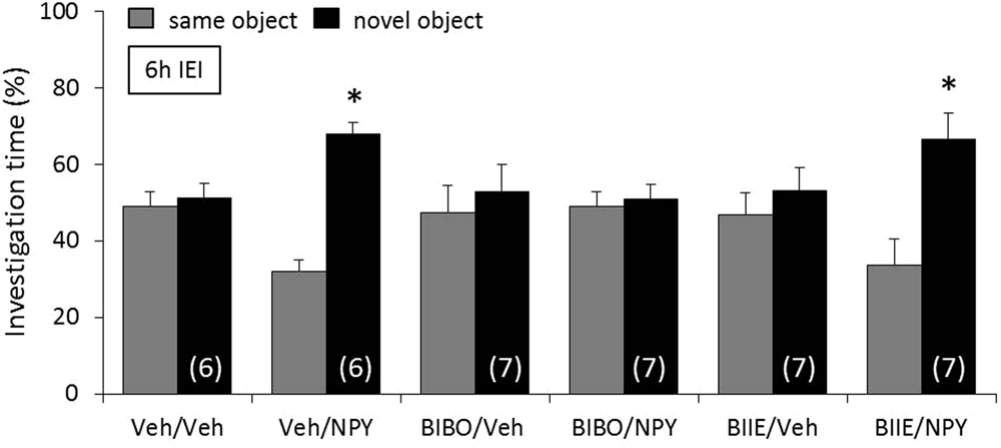
Neuropeptide Y (NPY) prolongs retention of non-social memory by acting on Y1, but not on Y2, receptors. Mice were infused icv with either vehicle (Veh; 2 µl), BIBO (2 nmol/2 µl), or BIIE (2 nmol/2 µl) immediately after the acquisition period of the object discrimination test. After 10 min, mice were infused again with either Veh (2 µl) or NPY (1 nmol/2 µl). Intact memory and discrimination abilities are reflected by an increased investigation of the *novel* versus the *same* object after an inter-exposure interval (IEI) of 6 h. Data represent means + SEM, and numbers in parentheses indicate group sizes. *p < 0.05 versus *same* object.

To determine whether the effects of NPY on non-social memory are mediated by the Y1 and/or Y2 receptors, mice were infused icv with either Veh, BIBO, or BIIE immediately after the acquisition period of the object discrimination test. After 10 min, mice were infused again with either Veh or NPY. Whereas Veh/Veh-treated mice showed impaired non-social memory after an IEI of 6 h, Veh/NPY-treated mice showed a higher investigation of the *novel* versus the *same* object, reflecting object discrimination and intact non-social memory (p < 0.05, Fig. 3). Both BIBO/Veh- and BIIE/Veh-treated mice showed impaired non-social memory. Whereas BIIE/NPY-treated mice showed intact non-social memory (p < 0.05, Fig. 3), BIBO/NPY-treated mice showed impaired non-social memory.

### NPY neurotransmission is not necessary for non-social and social memory acquisition

**Figure 4:**
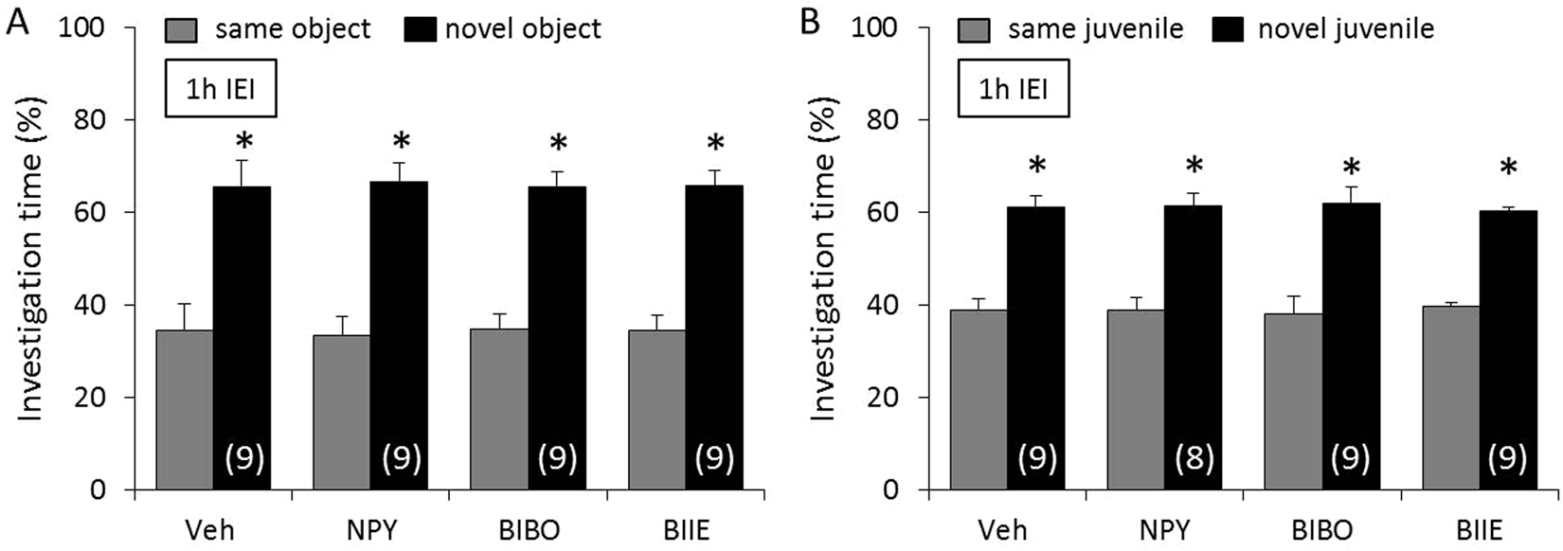
Neuropeptide Y (NPY) neurotransmission is not necessary for non-social and social memory acquisition. Mice were infused icv with either vehicle (Veh; 2 µl), NPY (1 nmol/2 µl), BIBO (2 nmol/2 µl), or BIIE (2 nmol/2 µl) 10 min before the acquisition period of the object (**A**) and social (**B**) discrimination test, respectively. Intact memory and discrimination abilities are reflected by an increased investigation of the *novel* versus the *same* stimulus after an inter-exposure interval (IEI) of 1 h. Data represent means + SEM, and numbers in parentheses indicate group sizes. *p < 0.05 versus *same* stimulus.

To determine whether NPY neurotransmission is necessary for non-social and social memory acquisition, mice were infused icv with either Veh, NPY, BIBO, or BIIE 10 min before the acquisition period of the object and social discrimination test, respectively. Mice showed a higher investigation of the *novel* versus the *same* object (p < 0.05, Fig. 4A) and juvenile (p < 0.05, Fig. 4B) after an IEI of 1 h, independent of treatment, reflecting intact non-social and social memory, respectively. As object and social investigation time during the acquisition period did not differ between the treatment groups (F(3,32) = 0.18; p = 0.91 for non-social memory and F(3,31) = 0.30; p = 0.82 for social memory), we can exclude possible confounding effects of NPY, BIBO, and BIIE on general motivation to investigate the stimuli.

### Y1 and Y2 receptor-mediated NPY neurotransmission is necessary for non-social, but not social, memory consolidation

**Figure 5:**
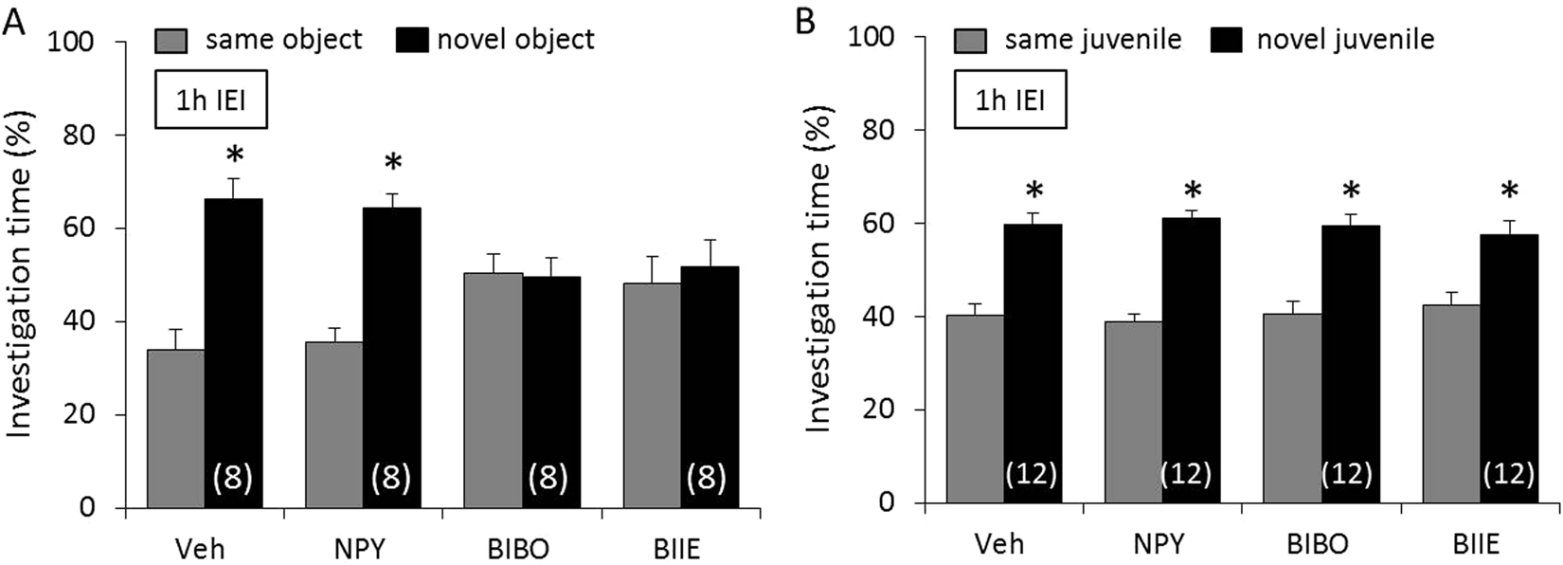
Y1 and Y2 receptor-mediated neuropeptide Y (NPY) neurotransmission is necessary for non-social, but not social, memory consolidation. Mice were infused icv with either vehicle (Veh; 2 µl), NPY (1 nmol/2 µl), BIBO (2 nmol/2 µl), or BIIE (2 nmol/2 µl) immediately after the acquisition period of the object (**A**) and social (**B**) discrimination test, respectively. Intact memory and discrimination abilities are reflected by an increased investigation of the *novel* versus the *same* stimulus after an inter-exposure interval (IEI) of 1 h. Data represent means + SEM, and numbers in parentheses indicate group sizes. *p < 0.05 versus *same* stimulus.

To determine whether NPY neurotransmission is necessary for non-social and social memory consolidation, mice were infused icv with either Veh, NPY, BIBO, or BIIE immediately after the acquisition period of the object and social discrimination test, respectively. Whereas Veh- and NPY-treated mice showed intact non-social memory after an IEI of 1 h (p < 0.05, Fig. 5A), BIBO- and BIIE-treated mice showed impaired non-social memory, reflected by similar investigation of the *same* and the *novel* object. In the social discrimination test, mice showed a higher investigation of the *novel* versus the *same* juvenile after an IEI of 1 h, independent of treatment, reflecting intact social memory (p < 0.05, Fig. 5B).

### Y1 and Y2 receptor-mediated NPY neurotransmission is necessary for both non-social and social memory retrieval

**Figure 6:**
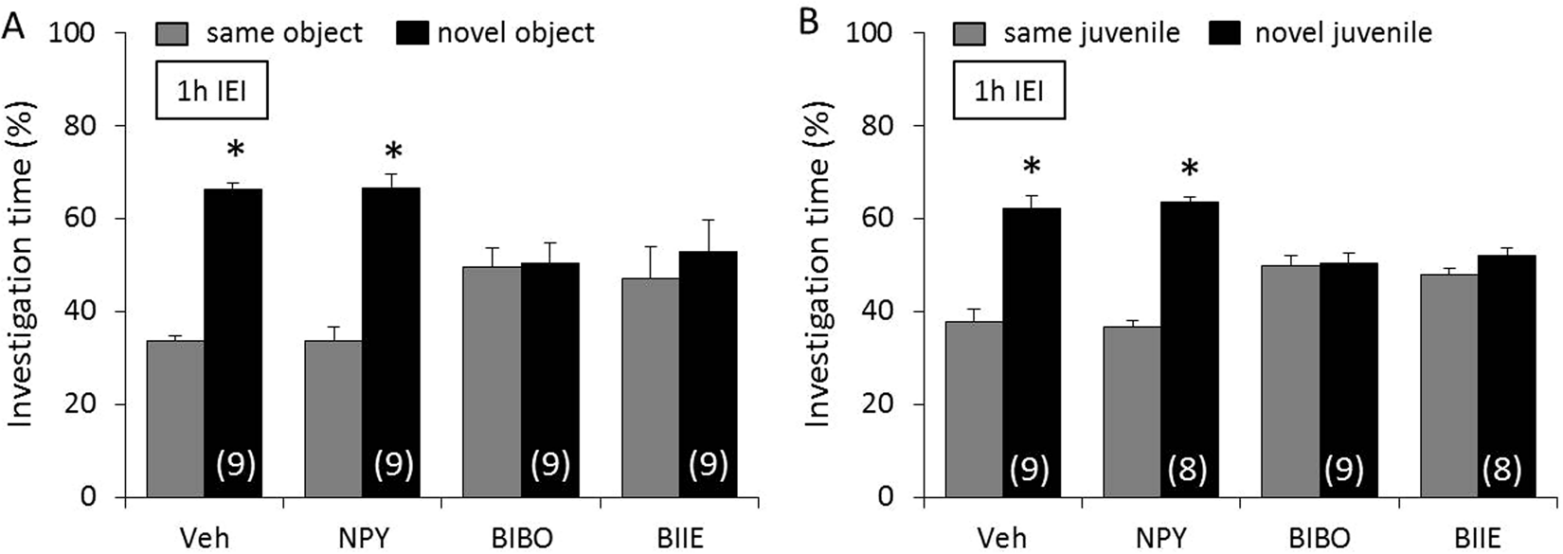
Y1 and Y2 receptor-mediated neuropeptide Y (NPY) neurotransmission is necessary for both non-social and social memory retrieval. Mice were infused icv with either vehicle (Veh; 2 µl), NPY (1 nmol/2 µl), BIBO (2 nmol/2 µl), or BIIE (2 nmol/2 µl) 10 min before the discrimination period of the object (**A**) and social (**B**) discrimination test, respectively. Intact memory and discrimination abilities are reflected by an increased investigation of the *novel* versus the *same* stimulus after an inter-exposure interval (IEI) of 1 h. Data represent means + SEM, and numbers in parentheses indicate group sizes. *p < 0.05 versus *same* stimulus.

To determine whether NPY neurotransmission is necessary for non-social and social memory retrieval, mice were infused icv with either Veh, NPY, BIBO, or BIIE 10 min before the discrimination period of the object and social discrimination test, respectively. Whereas Veh- and NPY-treated mice showed intact non-social (p < 0.05, Fig. 6A) and social (p < 0.05, Fig. 6B) memory after an IEI of 1 h, BIBO- and BIIE-treated mice showed impaired non-social and social memory, reflected by similar investigation of the *same* and the *novel* object and juvenile, respectively.

## Discussion

The present study demonstrates that icv NPY has the potential to prolong the retention of non-social, but not of social, memory in male mice by acting on Y1, but not on Y2 receptors. Furthermore, the central NPY system exerts differential effects on the sequential phases of non-social and social memory. As such, NPY did not disrupt the acquisition, consolidation, and retrieval of non-social and social memory at the dose used. Whereas Y1 and Y2 receptor-mediated NPY neurotransmission were not necessary for the acquisition of non-social and social memory, both Y1 and Y2 receptor-mediated NPY neurotransmission were necessary for consolidation of non-social memory and retrieval of both non-social and social memory.

We have previously shown that male mice and rats retain non-social (object) memory for at least 4 h, whereas social memory was retained for at least 1 h^27, 31, 32^, and could now confirm this effect. We could further show that non-social memory was impaired after 6 h, whereas social memory was impaired after 4 h. Icv administration of NPY prolonged the retention of non-social memory from 4 h to at least 6 h, while it was ineffective in prolonging the retention of social memory from 2 h to 4 h. A non-social memory-enhancing effect of NPY was previously suggested in studies showing that NPY counteracted the amnesic effects of anisomycin and scopolamine^21^, and attenuated the learning impairments induced by MK-801^25^. Interestingly, these memory-enhancing effects of NPY were similar to those of another neuropeptide, neuropeptide S, which was also shown to specifically prolong non-social, but not social, memory in male rats^31^. However, as distinct doses of NPY might differentially affect the retention of non-social and social memory, a possible limitation of this study is the use of a single dose of NPY to investigate its effects on retention of social memory. Furthermore, we cannot exclude that NPY prolongs the retention of social memory after shorter IEI (2,5 h or 3 h).

We could also show that NPY prolonged the retention of non-social memory by acting on Y1, but not on Y2, receptors, as the Y1 receptor antagonist BIBO, but not the Y2 receptor antagonist BIIE blocked this effect. This is in agreement with studies showing that [Leu31Pro34]NPY, a Y1 receptor agonist, counteracted the amnesic effects of MK-801^25^ and colchicine^33^ in non-social memory tasks such as passive avoidance and Morris water maze, respectively.

The anatomical substrates for the memory-enhancing effect of NPY in the object discrimination test (Fig. 2) still need to be identified, but may include the hippocampus, amygdala, and cortical regions as these brain regions express high levels of Y1 receptors^4^ and are involved in non-social memory tasks such as object discrimination^24^.

Given that many studies have shown differential drug effects on the sequential phases of memory^17, 22, 28, 32^, we next investigated the effects of NPY and its Y1 and Y2 receptors on the acquisition, consolidation, and retrieval of non-social and social memory. By using an IEI of 1 h where mice retain both non-social and social memory, we could show that NPY did not disrupt the acquisition, consolidation, and retrieval of non-social and social memory at the dose used. Whereas Y1 and Y2 receptor-mediated NPY neurotransmission were not necessary for the acquisition of non-social and social memory, both Y1 and Y2 receptor-mediated NPY neurotransmission were necessary for consolidation of non-social memory and retrieval of both non-social and social memory.

A lack of involvement of NPY and its Y1 and Y2 receptors in acquisition of memory is partly supported by the literature. As such, pre-training administration of NPY and PYY, a Y1 receptor agonist, did not affect acquisition of passive avoidance^22, 23^. Similarly, NPY knockout mice, Y2 receptor knockout mice, and conditional Y1 receptor knockout mice showed unaltered memory acquisition in passive avoidance, Morris water maze, and object discrimination tests^34–37^, supporting our findings. However, in cued and contextual fear conditioning, NPY was shown to impair acquisition by acting on Y1 receptors^15–17^, acting thereby as a resilience factor against exaggerated fear responses after stress and adverse events. Therefore, differences in the learning paradigms (e.g. aversive versus non-aversive) or in the type of conditioning (e.g. classical versus operant) may explain the partially different roles of the NPY system in memory acquisition. Given that the same dose of antagonists blocked the non-social memory-enhancing effect of NPY (Fig. 3), the consolidation of non-social memory (Fig. 5), and retrieval of both non-social and social memory (Fig. 6), a lack of involvement of Y1 and Y2 receptors in memory acquisition due to insufficient availability of the antagonist seems unlikely.

Our finding that NPY did not disrupt memory consolidation, while both Y1 and Y2 receptor antagonists impaired consolidation of non-social memory partly confirms previous studies in rats and mice. As such, both NPY knockout mice and NPY transgenic rats showed unaltered consolidation in non-social memory tasks such as passive avoidance^35, 38^, Morris water maze, and object discrimination tests^39^, respectively. Furthermore, conditional Y1 receptor knockout mice, Y2 receptor knockout mice, and wild-type mice infused with the Y1 receptor antagonist BIBP3266 showed impaired memory consolidation in these tests^33, 34, 37^, supporting our findings. Other studies, however, have shown that NPY and PYY, a Y1 receptor agonist, enhanced retention of active and passive avoidance memory^21, 22^.

We could also show that although NPY did not disrupt memory retrieval, both Y1 and Y2 receptor antagonists impaired retrieval of both non-social and social memory. The involvement of Y1 receptor in memory retrieval was previously suggested by Nakajima *et al*.^22^, who have shown that PYY, a Y1 receptor agonist, enhanced retrieval of passive avoidance memory. While no studies to date investigated the involvement of Y2 receptor in memory retrieval, the role of NPY is less clear. As such, NPY was shown to either impair retrieval of cued fear^19, 20^ or to enhance retrieval of active and passive avoidance^21^. As for memory acquisition, differences in the learning paradigms or the type of conditioning (e.g. classical versus operant) may explain the partially different roles of NPY in memory retrieval.

Interestingly, administration of the Y1 receptor antagonist BIBO resulted in the same behavioral profile as observed following administration of the Y2 receptor antagonist BIIE. Thus, both Y1 and Y2 receptor antagonists impaired consolidation of non-social memory and retrieval of both non-social and social memory. A previous study employing NPY knockout mice, Y1 receptor knockout mice, Y2 receptor knockout mice, and Y1/Y2 double knockout mice also suggested a synergistic effect of Y1 and Y2 receptors in the acquisition and consolidation of cued fear^40^. Similarly, the anxiolytic effects of NPY in the social interaction test seem to be mediated by both Y1 and Y2 receptors^11, 12^.

However, there are some possible limitations to the interpretation of our results. As such, the duration of memory retention in our experimental protocol is rather short and does not allow for a clear separation of acquisition, consolidation, and retrieval phases. As mentioned in the introduction, substances applied before acquisition might exert effects lasting long enough to affect subsequent processes like consolidation^2^. Neuropeptides have generally short half-lives and are active in the brain for less than 1 h. Although the exact half-lives of NPY, BIBO, and BIIE in the brain are not known yet, previous studies have shown that the half-lives of NPY and BIBP3226, a Y1 receptor antagonist, are quite short in the plasma, i.e. approximately 12 min^41^ and 20 min^42^, respectively. By using a short IEI (1 h), we cannot completely exclude that BIBO and BIIE administered immediately after the acquisition of non-social memory (Fig. 5) exerted effects which only affected consolidation but not retrieval. However, a separation of effects was possible in the case of social memory, where BIBO and BIIE impaired only retrieval but not consolidation of memory, suggesting that NPY, BIBO, and BIIE exerted effects lasting less than 1 h. A short-time bioavailability of NPY, BIBO, and BIIE might be also supported by the fact that pre-acquisition administration of BIBO and BIIE (Fig. 4) did not impair consolidation of non-social memory, which would start approximately 15–20 min after the infusion.

Taken together, we have shown that NPY has the potential to prolong the retention of non-social memory in male mice by acting on Y1, but not on Y2, receptors. Furthermore, while NPY did not disrupt acquisition, consolidation, and retrieval of memory, both Y1 and Y2 receptor-mediated NPY neurotransmission were necessary for consolidation of non-social memory and retrieval of both non-social and social memory. Thus, our study suggests that NPY specifically enhances non-social memory at the dose used and the brain NPY system differentially regulates the sequential phases of non-social and social memory.
